# Research progress of liposomal bupivacaine and its value in perioperative pain management

**DOI:** 10.1016/j.jpra.2025.06.022

**Published:** 2025-07-05

**Authors:** Hongli Sun, Yapeng Lu, Yingbin Wang

**Affiliations:** Department of Anesthesiology, Lanzhou University Second Hospital, No. 82 Cuiyingmen, Chengguan District, Lanzhou, Gansu Province, 730030, China

**Keywords:** Liposomal bupivacaine, Perioperative pain management, Regional nerve block

## Abstract

In current pain management practices, multimodal analgesia strategies centered on regional nerve blocks, combined with low-dose opioids and adequate nonsteroidal anti-inflammatory drugs, are considered the standard approach. However, the short-lived effectiveness of conventional local anesthetics leads to inadequate postoperative pain management, which substantially diminishes patient comfort and satisfaction during recovery periods. Prolonging the duration of local anesthetics has become a core focus of regional anesthesia research, with liposomal bupivacaine gaining attention due to its ultra-long-acting properties. This article aims to systematically explore the mechanism of action, safety, potential adverse reactions, and clinical applications of liposomal bupivacaine in nerve blocks and surgical procedures, providing a scientific basis for optimizing clinical analgesia strategies.

## Introduction

Burns and reconstructive surgeries often necessitate multiple procedures that involve cutting, reshaping, or repositioning tissues, which can result in significant pain, particularly at skin graft donor sites. This pain extends beyond mere physical discomfort; it can trigger a variety of psychosocial responses that substantially impact patients—delaying recovery, inducing anxiety and depression, leading to opioid dependence, increasing the risk of postoperative complications, affecting overall postoperative satisfaction, and raising healthcare resource consumption.

Under the guidance of Enhanced Recovery After Surgery (ERAS) protocols, optimized perioperative analgesia has become a critical objective in anesthesia.^1^ The strategic implementation of sustained-release local anesthetic liposomal bupivacaine (LB) aims to facilitate targeted pain modulation while minimizing reliance on opioids. Current analgesic protocols emphasize multimodal approaches, with peripheral nerve blockade serving as the foundational intervention. These interventions are strategically complemented by reduced opioid administration and evidence-based utilization of non-steroidal anti-inflammatory drugs (NSAIDs) to enhance pain control while mitigating pharmacological risks.

Contemporary strategies for prolonging regional anesthesia leverage multimodal pharmacological approaches, utilizing synergistic combinations of sodium channel blockers alongside adjunctive agents that target alpha-2 adrenergic, NMDA, and cytokine pathways. This multifaceted approach facilitates improved opioid stewardship in perioperative pain management. In this context, extending the duration of local anesthetic analgesia remains a key research focus in regional anesthesia. Long-acting local anesthetics, particularly LB, have garnered significant attention from researchers.

LB is a sustained-duration regional anesthetic that received FDA clearance in 2011, specifically indicated for surgical site analgesia through single-injection peripheral nerve blockade. It has demonstrated extended therapeutic efficacy in postoperative pain control.^2^ While conventional local anesthetics effectively relieve pain, their duration of action is relatively short, often requiring additional postoperative opioids or other analgesics, which may heighten the risk of drug-related side effects and dependency.

To prolong postoperative analgesia, anesthesiologists typically employ continuous catheter-based infusion or adjuvants such as opioids, epinephrine, steroids, or α₂ receptor agonists.^3^ However, while continuous nerve blocks provide sustained analgesia, they may also increase the risks of trauma, infection, nerve injury, and procedural complexity. Additionally, the use of adjuvants comes with potential adverse reactions, and their reliability in extending analgesia is uncertain. These methodologies can also result in higher hospitalization costs and diminish patient comfort and satisfaction.

As a long-acting local anesthetic, LB offers superior pain management for surgeries necessitating prolonged analgesia and demonstrates a favorable safety profile.

### Mechanism of action

LB is an innovative long-acting local anesthetic that utilizes liposomal encapsulation technology to prolong the release of bupivacaine, providing effective pain relief for up to 72 h.^4^^,^^5^ Its primary mechanism of action involves the reversible blockade of sodium ion channels, which inhibits nerve signal transmission and achieves analgesic effects.

As a drug delivery system, liposomes offer several advantages:

1. Prolonged Release: Controlled release of the drug ensures long-lasting analgesia.
2. Reduced Toxicity: Slower absorption into the bloodstream decreases the risk of cardiotoxicity and central nervous system toxicity.
3. Enhanced Targeting: Increased local concentration of the drug at the target site improves analgesic efficacy.
4. Minimized Tissue Irritation: Alleviation of local inflammatory responses reduces tissue irritation.

### Safety, potential toxicity and management

The most common adverse events associated with LB include nausea, vomiting, and constipation. Viscusi et al. conducted a comprehensive pooled analysis of safety data regarding the localized application of LB in various surgical procedures, including hemorrhoidectomy, bunionectomy, breast augmentation, total knee arthroplasty, and hernia repair.^6^ The analysis revealed no statistically significant variation in the occurrence of adverse events among patients treated with LB compared to those administered bupivacaine or placebo. Similarly, a study by Freitas de Lima et al. showed that LB significantly reduces toxicity and adverse reactions compared to conventional local anesthetics.^7^

The maximum recommended dose of LB for adults is 4 mg/kg (up to 266 mg); however, its use is contraindicated in pregnant patients. The risk of local anesthetic toxicity primarily depends on patient-related factors, drug properties, and the site of administration. While most patients can tolerate relatively high doses of local anesthetics, the risk of toxicity is greater in very young or elderly individuals.^8^

Neonates and infants have lower plasma protein levels and reduced binding capacity for local anesthetics, leading to a higher concentration of free drug in the bloodstream and an increased risk of clinical toxicity. In elderly patients, underlying conditions such as hepatic, renal, or cardiac dysfunction may impair drug metabolism and excretion, further elevating the risk of toxicity from local anesthetics.^9^^,^^10^

Bupivacaine may cause vasodilation, which can accelerate systemic absorption and increase toxicity risk. However, LB mitigates this effect by releasing bupivacaine slowly, thus reducing the rate and overall amount of the drug entering systemic circulation and lowering its toxic potential. Currently, there are no published reports of toxicity specifically associated with LB, which may be attributed to its limited clinical use thus far.

In the event of local anesthetic systemic toxicity (LAST), management should adhere to established protocols applicable to other local anesthetics, which include the immediate administration of intravenous lipid emulsion therapy. It is critical to avoid combining LB with other local anesthetics such as lidocaine, as this could lead to a rapid release of bupivacaine, thereby heightening the risk of severe cardiovascular and neurological toxicity.^10^ Clinicians are advised to wait a minimum of 20 min after administering lidocaine before using LB and to refrain from administering other local anesthetics during this period.

Furthermore, reports have indicated fetal bradycardia and even death following the use of LB in obstetric paracervical nerve blocks.^11^ Although spinal anesthesia is frequently employed for lower limb surgeries, the LB product label advises against its use for spinal administration. This recommendation may stem from a lack of safety and toxicity data for intrathecal use, as well as concerns regarding unintentional subarachnoid administration during epidural procedures.

Local anesthetic systemic toxicity is a critical and potentially fatal medical emergency. Clinicians must be thoroughly familiar with its risk factors, clinical manifestations, and management strategies prior to the administration of any local anesthetic.

### Clinical applications

LB, as a novel long-acting local anesthetic, has demonstrated unique advantages in postoperative analgesia in recent years. By employing liposomal sustained-release technology, it extends the duration of drug action and has shown analgesic benefits across various surgical procedures. However, some controversies surrounding its application persist in certain fields.

In the study by Jonas et al., titled "Perioperative Inpatient Opioid Consumption Following Autologous Free-Flap Breast Reconstruction: An Examination of Risk and Patient-Reported Outcomes," the use of LB was associated with reduced opioid consumption in patients undergoing autologous breast reconstruction.^12^ Similarly, Farzin et al. conducted a retrospective analysis that found LB infiltration at reconstructive skin graft donor sites significantly decreased postoperative pain scores in adolescent and young adult burn patients.^13^ Additionally, Christina et al. reported that patients who received LB experienced less postoperative donor site pain and reported the donor site to be less bothersome, without major complications.^14^ These findings suggest that LB may be a safe and promising agent for prolonging postoperative analgesia and minimizing donor site pain.

Some studies have reported that local infiltration of LB after tooth extraction significantly reduces postoperative pain scores and decreases opioid consumption.^15^ For example, Patel et al. performed a retrospective cohort study involving 38 patients who received anterior iliac crest bone grafts.^16^ The results indicated that patients receiving local infiltration of LB experienced significantly lower average pain scores within 24 h post-surgery compared to those receiving 0.25 % bupivacaine, accompanied by a notable reduction in opioid consumption.

Kolade et al. conducted a meta-analysis evaluating the effectiveness of LB in shoulder procedures, including shoulder arthroplasty and rotator cuff repair.^17^ They found that LB offered significantly improved postoperative pain relief compared to conventional local anesthetics, particularly by extending the duration of analgesia and decreasing opioid consumption. Therefore, in the context of reducing dependence on opioids and their associated side effects, LB demonstrates potential clinical advantages in postoperative pain management for shoulder surgeries.

Lee et al. investigated the application of LB in sternotomy, particularly for intercostal nerve block, through a randomized controlled trial.^18^ They observed that patients in the LB group reported significantly lower pain scores at both 24 and 48 h post-surgery compared to those who received conventional bupivacaine, with a notably prolonged duration of pain relief within the first 48 h postoperatively. The LB group also required significantly fewer opioids, underscoring LB's ability to reduce postoperative opioid use and, consequently, mitigate the risks of side effects and dependency. Minor side effects, such as nausea and vomiting, occurred at similar rates in both groups, and no severe adverse events were reported, suggesting that LB has a favorable safety profile.

Meyer et al. examined the effectiveness of LB versus standard bupivacaine for incision site infiltration analgesia in open gynecologic surgeries, with a focus on its role within the Enhanced Recovery Pathway (ERP).^19^ This study indicated that LB provided significantly better incision infiltration analgesia than standard bupivacaine, resulting in a more prolonged analgesic effect, reduced postoperative opioid use, and accelerated patient recovery, ultimately shortening hospital stays.^20^ Malige et al. carried out a randomized controlled trial involving 100 patients and found that LB significantly reduced postoperative pain following total knee arthroplasty (TKA), led to shorter hospital stays, and decreased in-hospital opioid consumption.^21^

Contrastingly, some studies have reported different outcomes. Bagsby et al. conducted a retrospective cohort study on 150 TKA patients receiving LB for local infiltration analgesia and found no improvement in pain control based on multimodal analgesia protocols.^22^ Similarly, Quaye et al. prospective study indicated that while LB significantly reduced opioid use after TKA, the differences in postoperative visual analog scale (VAS) scores were not statistically significant.^23^

Hamilton et al. compared LB combined with bupivacaine to bupivacaine alone for periarticular analgesia in knee arthroplasty patients in a randomized clinical trial, finding no differences in postoperative recovery or pain scores.^24^ This suggests that the analgesic efficacy of LB in TKA may depend on the design of preoperative analgesia protocols, necessitating a comprehensive evaluation in clinical scenarios.

Moreover, Chen et al. found that LB did not demonstrate superior analgesic effects compared to standard bupivacaine across various surgical types.^25^ Jin et al. reported no additional benefits of LB in knee arthroplasty, laparoscopic urological surgery, and breast surgery.^26^ Chitty et al. applied LB for local infiltration analgesia after hemorrhoidectomy and noted significantly lower early postoperative pain scores compared to the bupivacaine group; however, subsequent pain scores and the use of rescue analgesics showed no differences.^27^

A meta-analysis by Nguyen et al., which included 27 trials with 2122 patients, revealed that LB reduced resting pain scores at 24, 48, and 72 h post-surgery. Overall, these studies indicate that the analgesic effects of LB remain highly controversial in clinical practice, highlighting the urgent need for further research to clarify its value in clinical applications.^28^

### Disadvantages

LB, being a liposome-encapsulated drug, has a slower onset of action compared to traditional bupivacaine. To address immediate analgesic needs, it can be combined with hydrochloride bupivacaine. Furthermore, the complex production process of LB leads to a substantially higher cost than that of standard bupivacaine. Current studies indicate variable efficacy, which limits its widespread clinical application.

The maximum single dose of LB is restricted, potentially necessitating multiple injections or combinations with other drugs, thereby increasing complexity. Injection sites may experience mild swelling, pain, or inflammation; although the incidence is low, it still requires attention. When used in conjunction with other local anesthetics, there is an increased risk of compounded toxicity, necessitating strict adherence to dosing intervals (e.g., a minimum of 20 min).

While LB is suitable for postoperative scenarios requiring long-lasting analgesia, its associated costs, onset time, and specific patient needs must be carefully considered. Clinical use should adhere to established guidelines to ensure safety and efficacy.

## Conclusions

In summary, as the concept of ERAS evolves, perioperative pain management has emerged as a critical focus for anesthesiologists. Traditional local anesthetics generally provide pain relief for <12 h, often necessitating opioid administration for prolonged pain management.

However, the side effects associated with opioids—including respiratory depression and the risk of addiction—as well as concerns related to patient satisfaction have driven the adoption of "opioid-sparing" strategies.

LB, as an ultra-long-acting local anesthetic (with a duration of action of 72–96 h), extends the effectiveness of regional blocks and has become a pivotal technology in achieving opioid-sparing within multimodal analgesia. LB has revolutionized postoperative pain management through its sustained-release technology, significantly reducing opioid dependence and enhancing patient comfort.^29^

Nonetheless, the clinical application of LB must weigh its costs, onset time, and individual patient needs.^30^ Future research should prioritize multicenter, large-sample studies to validate its long-term benefits, optimize dosing regimens, and explore strategies for integration into the ERAS pathway.

## Ethics approval

Not required.

## Funding

This work was supported by General Program of the National Health Commission of the People’s Republic of China under Grant WKZX2024CX301208.

## Conflicts of interest

The authors declare that they have no known competing financial interests or personal relationships that could have appeared to influence the work reported in this paper.

## References

[1] Guo C., Lou F., Wu J., Zhang J. (2024). ERAS-based anesthetic management of patients undergoing abdominal-based free flap breast reconstruction: A narrative review. JPRAS Open.

[2] Zhang Q.D., Duan Q.Y., Tu J., Wu F.G. (2023). Thrombin and thrombin-incorporated biomaterials for disease treatments. Adv Healthc Mater.

[3] Terres M.T., Machado Assis M.L., Lombardi R.A., Balthazar da Silveira C., Amaral S. (2025). Adding dexmedetomidne to intra-articular local anesthetics results in prolonged analgesia after knee arthroscopy: A systematic review and meta-analysis. Arthroscopy.

[4] Coppens S.J.R., Zawodny Z., Dewinter G. (2019). In search of the holy grail: Poisons and extended release local anesthetics. Best Pract Res Clin Anaesthesiol.

[5] Urman R., Malik O., Kaye A., Kaye A., Belani K. (2017). Emerging roles of liposomal bupivacaine in anesthesia practice. J Anaesthesiol Clin Pharmacol.

[6] Viscusi E.R., Sinatra R., Onel E., Ramamoorthy S.L. (2014). The safety of liposome bupivacaine, a novel local analgesic formulation. Clin J Pain.

[7] Freitas de Lima F., da Silva B.B., Oliveira J.D. (2021). Prolonged anesthesia and decreased toxicity of enantiomeric-excess bupivacaine loaded in ionic gradient liposomes. Int J Pharm.

[8] Malige A., Yeazell S., Ng-Pellegrino A., Carolan G. (2020). Risk factors for complications and return to the emergency department after interscalene block using liposomal bupivacaine for shoulder surgery. J Shoulder Elbow Surg.

[9] Waldinger R., Weinberg G., Gitman M. (2019). Local anesthetic toxicity in the geriatric population. Drugs Aging.

[10] Aggarwal N. (2017). Local anesthetics systemic toxicity association with exparel (bupivacaine liposome)- a pharmacovigilance evaluation. Expert Opin Drug Saf.

[11] Hussain N., Brull R., Sheehy B. (2021). Perineural liposomal bupivacaine is not superior to nonliposomal bupivacaine for peripheral nerve block analgesia. Anesthesiology.

[12] Nelson J.A., Polanco T.O., Shamsunder M.G. (2021). Perioperative inpatient opioid consumption following autologous free-flap breast reconstruction patients: An examination of risk and patient-reported outcomes. Ann Surg Oncol.

[13] Sadeq F., DePamphilis M.A., Dabek R.J. (2022). Evaluation of liposomal bupivacaine infiltration at reconstructive skin graft donor sites in adolescent and young adult burn patients: a retrospective analysis. Burns.

[14] Artz C., Ward M.A., Miles M.V.P. (2021). Intraoperative liposomal bupivacaine for skin graft donor site analgesia: a retrospective cohort study. Burns.

[15] Lieblich S.E., Misiek D., Olczak J., Fleck H., Waterman F. (2021). A retrospective cross-sectional study of the effect of liposomal bupivacaine on postoperative opioid prescribing after third molar extraction. J Oral Maxillofacial Surg.

[16] Patel R.A., Jablonka E.M., Rustad K.C. (2019). Retrospective cohort-based comparison of intraoperative liposomal bupivacaine versus bupivacaine for donor site iliac crest analgesia during alveolar bone grafting. J Plast Reconstruct Aesthet Surg.

[17] Kolade O., Patel K., Ihejirika R. (2019). Efficacy of liposomal bupivacaine in shoulder surgery: a systematic review and meta-analysis. J Shoulder Elbow Surg.

[18] Lee C.Y., Robinson D.A., Johnson C.A. (2019). A randomized controlled trial of liposomal bupivacaine parasternal intercostal block for sternotomy. Ann Thorac Surg.

[19] Meyer L.A., Corzo C., Iniesta M.D. (2021). A prospective randomized trial comparing liposomal bupivacaine vs standard bupivacaine wound infiltration in open gynecologic surgery on an enhanced recovery pathway. Am J Obstet Gynecol.

[20] Little A., Brower K., Keller D., Ramshaw B., Janis J.E. (2019). A cost-minimization analysis evaluating the use of liposomal bupivacaine in reconstructive plastic surgery procedures. Plast Reconstruct Surg.

[21] Malige A., Pellegrino A.N., Kunkle K. (2022). Liposomal bupivacaine in adductor canal blocks before total knee arthroplasty leads to improved postoperative outcomes: A randomized controlled trial. J Arthroplasty.

[22] Bagsby D.T., Ireland P.H., Meneghini R.M. (2014). Liposomal bupivacaine versus traditional periarticular injection for pain control after total knee arthroplasty. J Arthroplasty.

[23] Quaye A., McAllister B., Garcia J.R. (2024). A prospective, randomized trial of liposomal bupivacaine compared to conventional bupivacaine on pain control and postoperative opioid use in adults receiving adductor canal blocks for total knee arthroplasty. Arthroplasty.

[24] Hamilton T.W., Athanassoglou V., Trivella M. (2016). Liposomal bupivacaine peripheral nerve block for the management of postoperative pain. Cochrane Database Systemat Rev.

[25] Chen J-J, Wu Y-C, Wang J-S, Lee C-H (2023). Liposomal bupivacaine administration is not superior to traditional periarticular injection for postoperative pain management following total knee arthroplasty: A meta-analysis of randomized controlled trials. J Orthop Surg Res.

[26] Jin Z.S., Ding O., Islam A., Li R., Lin J. (2021). Comparison of liposomal bupivacaine and conventional local anesthetic agents in regional anesthesia: A systematic review. Anesth Analg.

[27] Chitty L., Ridley B., Johnson B. (2022). Liposomal compared to 0.25% bupivacaine in patients undergoing hemorrhoidectomy: A pre- and post-implementation quality improvement evaluation. J Clin Anesth.

[28] Nguyen A., Grape S., Gobbetti M., Albrecht E. (2023). The postoperative analgesic efficacy of liposomal bupivacaine versus long-acting local anaesthetics for peripheral nerve and field blocks. Eur J Anaesthesiol.

[29] Sanghvi A.P., Klumb I., Kanani C., Karmarkar A., Kazior M. (2025). Efficacy of pectoralis nerve blocks I & II with liposomal bupivacaine in patients undergoing elective breast reduction procedures: a retrospective study. JPRAS Open.

[30] Lombana N.F., Beard C., Mehta I.M. (2024). The effect of a local anesthetic cocktail in a serratus anterior plane and PECS 1 block for implant-based breast reconstruction. JPRAS Open.

